# Human Decision Making Based on Variations in Internal Noise: An EEG Study

**DOI:** 10.1371/journal.pone.0068928

**Published:** 2013-07-01

**Authors:** Sygal Amitay, Jeanne Guiraud, Ediz Sohoglu, Oliver Zobay, Barrie A. Edmonds, Yu-Xuan Zhang, David R. Moore

**Affiliations:** Medical Research Council Institute of Hearing Research, Nottingham, United Kingdom; Radboud University Nijmegen, Netherlands

## Abstract

Perceptual decision making is prone to errors, especially near threshold. Physiological, behavioural and modeling studies suggest this is due to the intrinsic or ‘internal’ noise in neural systems, which derives from a mixture of bottom-up and top-down sources. We show here that internal noise can form the basis of perceptual decision making when the external signal lacks the required information for the decision. We recorded electroencephalographic (EEG) activity in listeners attempting to discriminate between identical tones. Since the acoustic signal was constant, bottom-up and top-down influences were under experimental control. We found that early cortical responses to the identical stimuli varied in global field power and topography according to the perceptual decision made, and activity preceding stimulus presentation could predict both later activity and behavioural decision. Our results suggest that activity variations induced by internal noise of both sensory and cognitive origin are sufficient to drive discrimination judgments.

## Introduction

Perceptual decision making is one of the most important tasks performed by the brain. Determining whether an event has occurred (detection) or whether an event is different from other events (discrimination) can mean the difference between life and death if, for example, the event is a snapping twig which could herald the arrival of prey or predator. The brain must therefore have evolved a sophisticated and rapid mechanism for making decisions based on input from the external world. Physical stimuli have certain, well-defined properties when they arrive at the sensory organs, but they are then transduced and transmitted along pathways that have multiple synaptic way-stations where information can be delayed, distorted or influenced by descending, efferent activity. The accumulation of these effects is referred to as ‘internal noise’ [1], and includes the stochastic nature of neuronal firing (e.g. [2]), the internal state of the organism (e.g. arousal), or fluctuations in attention [3]. Percepts, or internal stimulus representations, are therefore imperfect representations of the physical stimulus, and physically identical stimuli can elicit variable percepts. The decision-making mechanism must act on the perceptual evidence available to achieve the organism’s goals. When stimuli are suprathreshold, behavioural decisions accurately reflect the internal representations, which in turn accurately reflect the properties of the physical stimuli (e.g. [4,5]). The fidelity of internal representations decreases near threshold, however, as the external signal becomes weaker. As the relative contribution of the internally-generated noise to the percept increases, decision-making becomes increasingly prone to errors.

In the extreme case, where discriminable variance of the external stimulus is absent, the percept is defined by the internal noise, allowing researchers to probe the limits of the decision-making mechanism and assess the influence internal noise sources have on its functioning. For example, when stimulated by a dynamic random dot visual display that lacked a coherent motion signal, the variability in firing rates of individual motion-sensitive neurons in cortical area MT correlated with a monkey’s obligatory perceptual choices of ‘motion’ direction [6]. Likewise, in a binocular rivalry paradigm, Dodd and colleagues [7] showed that neuronal firing in area MT correlated with the monkey’s reported depth sensation in the absence of binocular disparity (depth cue) in the visual display. In humans, functional magnetic-resonance imaging (fMRI) showed that activation of primary visual cortex (V1) changed during constant stimulation when the reported perception of a dichoptic image fluctuated between the two eyes in a binocular rivalry paradigm [8]. And, in an fMRI study of facial expression perception (fear or disgust), the network of areas activated in response to a neutral face correlated with the reported choice [9]. In the animal work the modulation in single neuron firing rates appeared within approximately 50 ms of stimulus onset, leading to the conclusion that they were driving the decision process rather than resulting from it [6]. While fMRI lacks sufficient temporal resolution to make this distinction possible, modeling evidence (e.g. [10,11]) indicates that noise can drive percepts.

More recently, Bernasconi and colleagues [12] have used electroencephalography (EEG) to show that perceptual decisions in an auditory discrimination task can be predicted by topographic differences in brain activity as early as 100 ms following stimulus onset. The finer temporal resolution afforded by EEG thus shows decision-predicting differences occur within a similar time-scale to the animal studies. However, the topographic modulation shown by these results differs in kind from the changes in neuronal firing rates in more local-area networks observed in animals. Modeling work based on signal detection theory (SDT) shows internal noise can cause large enough variations to drive perceptual decisions [13,14]. These models are based on overall activity differences between decision options rather than topographic variation. Specifically, the work of Micheyl et al. [13] was based on behavioral data we collected during a 3-interval, 3-alternative forced choice paradigm requiring listeners to pick the ‘odd-one-out’ of 3 identical tones [15]. We hypothesized that if internal noise adds a randomly fluctuating component (or components) to brain activity which is unrelated to the task, the neural response to the three identical tones will not be identical. By asking participants to pick the odd-one-out of these identical tones, we ask them to make a decision based on those fluctuations and can therefore quantify the difference in activity that results in a behavioural decision. We predicted that early stimulus-related activity will reflect these fluctuations, and later activity differences will reflect the processes leading up to and including decision making based on them. We present evidence from human EEG, recorded during the same ‘odd-one-out’ task, that shows early differences, both in overall activity and in topography of cortical activation predict perceptual decision-making.

## Experimental Methods

### Participants

Twenty right-handed listeners with normal hearing (≤ 20 dB HL bilaterally, 0.5-4 kHz; British Society of Audiology standard, method A [16]), aged 18-37 years (mean age 24.7; 6 males, 14 females), participated in the study.

Data from one listener were incomplete due to a technical fault and were excluded. In two other listeners an N1 peak could not be reliably identified using automated peak picking (see details below) and their data were also excluded.

### Ethics Statement

The research protocol was approved by the National Research Ethics Service Committee East Midlands - Nottingham 1, and written informed consent was obtained from all participants.

### Behavioral task

Listeners performed an auditory discrimination task. They were instructed to choose the odd-one-out of three consecutive tones, which, unbeknownst to them, were physically identical. They were told that the task will be very difficult. They were not instructed on which stimulus dimension to attend or to base their decision, but having successfully completed a frequency discrimination task with real differences just prior to EEG data collection we assume they were attending to frequency differences. The perceived differences engendered by this paradigm are quite salient, and only one of the twenty subjects reported hearing no differences in the sounds during post-experimental debriefing. Taken together with evidence from previous training studies using identical stimuli and similar task paradigms [15,17], which induced comparable learning to training with actual frequency differences, we believe listeners were attentive and engaged with the task.

**Figure 1:**
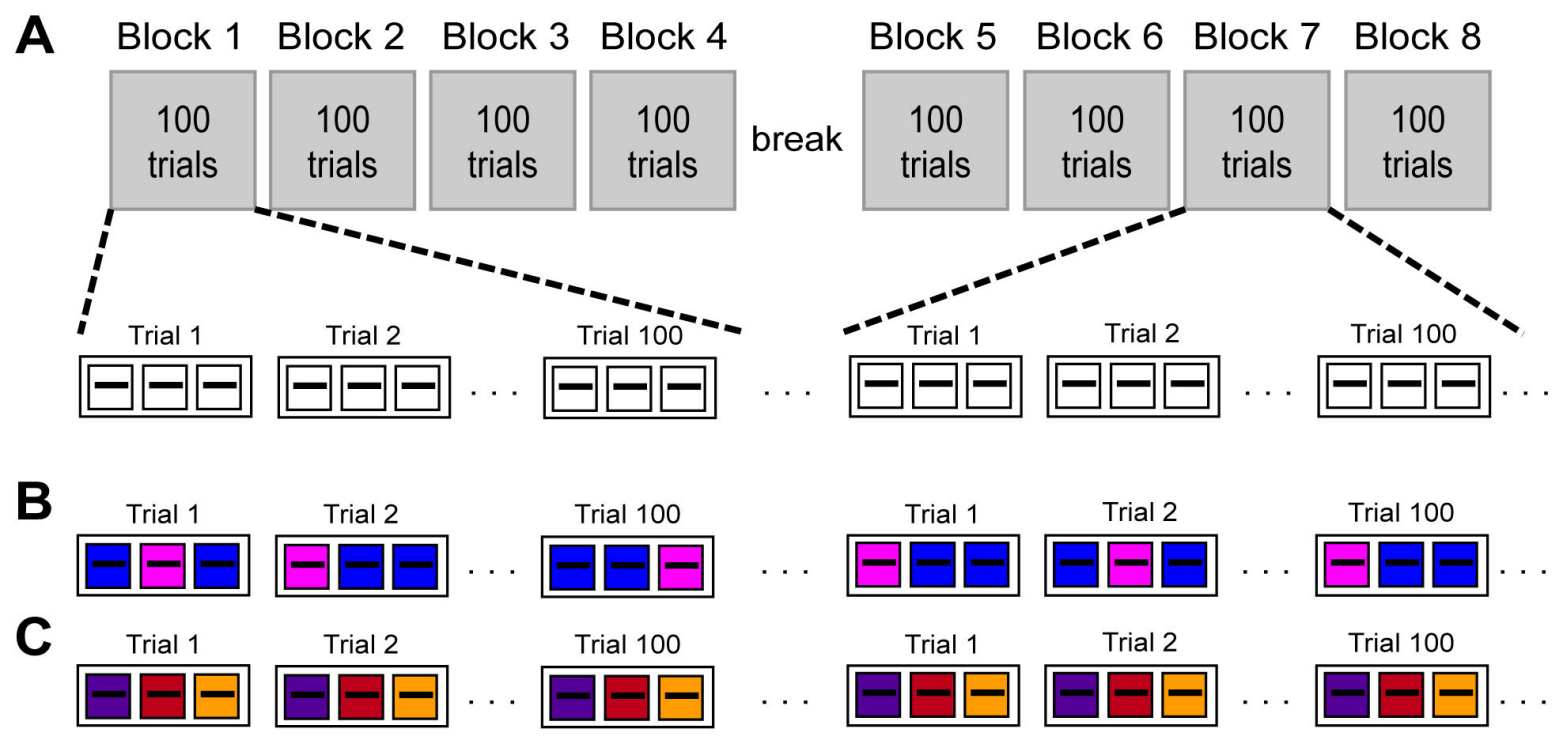
Experimental design and main factors in the statistical model. (A) Testing was divided into eight blocks of 100 trials with brief pauses between runs and a longer, 10-minute rest break between block 4 and 5. Each trial consisted of three stimulus intervals, each containing an identical 1-kHz tone. The tones were 100 ms long, separated by a 506 ms silent inter-stimulus interval (plus jitter; see Methods section) to a total trial-duration of approximately 1320 ms. The statistical model incorporated the following factors: (B) Activity produced by stimuli ‘chosen’ as different (magenta), indicated by a button press, was compared to that produced by ‘non-chosen’ stimuli (blue). (C) Activity was compared for interval 1 (purple), 2 (red) and 3 (orange) for each trial. The same color scheme is used for the traces in subsequent figures.

Listeners performed this task over 8 blocks of 100 trials each with short breaks between blocks and a longer break half way through the recording session (Figure 1A). Tones were presented diotically at 80 dB SPL through Sennheiser HD-25-I headphones. Each tone was 100-ms long, with 10-ms rise-fall times, and the tones were separated by a silent interval that was 506-ms long with an additional jitter. The jitter Poisson distributed (mean 3.6 ms) and was introduced to avoid N1-P2 complex amplitude increases due to α-band activity entraining to the temporal regularity within trials (see 18).

The task was presented as a computer game using custom software [19]. The 3 stimulus intervals were represented by three ‘cartoon’ characters of identical shape and size, presented at equal distances horizontally across the computer monitor (see screenshot in Figure S1 online). The characters were animated to open their mouths to coincide with the tones being played, each in turn from left to right. Once all the tones had been played, listeners responded without time limit using a three-button response box placed under three fingers of their right hand. Trials were initiated 700 ms (plus the same jitter described above) after the response to the previous trial. Following the feedback paradigm we used previously to induce perceptual learning with identical tones [15], positive visual feedback was provided during the inter-trial period on one third of the trials; the interval regarded as “correct” was randomly determined by the software and the character associated with that interval was briefly animated. Listeners were instructed to move as little as possible during blocks and to keep their eyes open, but were not asked to fixate. The experiment was carried out in a double-walled sound-attenuating booth.

### Electrophysiological recording

Auditory evoked potentials were recorded with a 32-channel EEG amplifier system (BrainAmp DC, Brain Products, Gilching, Germany) and an electrode cap (“infracerebral” cap, Easy Cap, Herrsching-Breitbrunn, Germany). The cap was fitted with 31 Ag/AgCl ring electrodes in a quasi-equidistant arrangement, which covers the largest possible area. Data were recorded continuously with a sampling rate of 500 Hz. They were analogue-filtered online between 0.02 and 250 Hz. Skin to electrode impedances were maintained below 5 kΩ. The ground electrode was placed on the midline of the forehead and the recording reference was the midline central electrode (Cz). Subjects were seated in a comfortable chair, with the response box on their lap under their right hand. They were instructed to relax, and move as little as possible.

### Electrophysiological analysis

The raw EEG data were preprocessed with the EEGLAB toolbox [20], which runs under MATLAB (Mathworks, http://www.mathworks.com), using methods adapted from Briley and colleagues [21]. The data were (i) bandpass-filtered between 0.1 and 35 Hz using a 32^nd^-order zero-phase Butterworth filter (16^th^ order applied forward and backward), (ii) channels with excessive noise (> 5 µV^2^/Hz at frequencies > 15 Hz) were removed on a block-by-block basis to avoid introducing the noise across all electrodes when re-referencing (these were generally back channels O_1/2_ or PO9/10, with occasionally T7/8, and amounted to 1.4% of total, with only 0.1% having more than 3 channels removed in the same block, maximum 5 on 2 blocks), (iii) re-referenced to the common average reference and (iv) epoched from -100 to 500 ms relative to stimulus onset. (v) Epochs with non-stereotypic artefacts were rejected automatically using the joint probability function in EEGLAB, which treats the presence of uncommonly large potentials across many electrodes as artefactual (vi). Stereotypic artefacts (e.g. electroocular activity) were eliminated by applying an independent components analysis (ICA) based on the extended infomax algorithm [22] and rejecting artefactual components by manually inspecting the components’ activity time courses, field maps and event-related average waveforms. These comprised 13.8% of all components: 5.2% eye-blinks, 2.3% lateral eye movements, 2.4% excessive α activity, 3.1% electrode pop-outs, 0.1% ECG and the remaining 0.8% residual noise (vii). Previously removed channels and Cz were reconstructed, and only then (viii) the data were separated into ‘chosen’ and ‘non-chosen’ epochs based on response button press to each trial, and (ix) baseline corrected to the 100 ms period preceding tone onset. In total, 15.3% of interval 1, 10.8% of interval 2, and 11.8% of interval 3 epochs were rejected during pre-processing. Of the remaining, ‘chosen’ epochs comprised 37.6% (4331 epochs) of interval 1 data, 33.7% (4085 epochs) of interval 2 data and 28.7% (3444 epochs) of interval 3 data. ‘Non-chosen’ epochs comprised 62.4% (7188 epochs), 66.3% (8049 epochs) and 71.3% (8556 epochs) of intervals 1, 2 and 3, respectively. These proportions accurately reflect the behavioral choices in each interval based on the entire dataset (see analysis of behavioral results in the Results section).

### Statistical analysis

We identified the N1 peaks for each listener as the minima in the 70-130 ms post-stimulus time window and the P2 as the maxima in the 150-210 ms post-stimulus time window. For the purpose of statistical analysis, we used the mean peak amplitudes over 25 ms (13 time-points) and 30 ms (16 time-points) for the N1 and P2, respectively, corresponding to the grand-average peak width at the half-power point (-3 dB gain, or 0.707 relative to the peak). Using the mean amplitude alleviates the SNR-difference due to non-chosen intervals being twice as numerous as chosen ones [23]. To avoid potential baseline differences between chosen and non-chosen responses we used the N1-P2 difference rather than absolute mean peak amplitudes (see 21 for discussion of the advantages of this method). The N1 and P2 are generally largest over fronto-central electrodes (Fz, FC_1/2_ and Cz). In choosing both peak time-intervals and electrode sites for analysis we followed Ben-David et al. [24] who used similar stimuli. We used a repeated-measures ANOVA to investigate the effect of response (chosen, non-chosen; Figure 1B), electrode, and interval position in the trial (1^st^, 2^nd^ or 3^rd^; Figure 1C) on N1-P2 mean peak amplitude differences. The N1-P2 difference was also analysed over lateral electrodes, using a repeated-measures ANOVA with hemisphere (left vs. right), electrode (TP9/10, P7/8 and PO9/10, going from anterior to posterior), interval (1^st^, 2^nd^ or 3^rd^), and choice (chosen, non-chosen) as within subject factors. Effects were considered significant for p < 0.05, Greenhouse–Geisser corrected where the data were not spherical.

To examine the effect of perceptual decision (whether a tone was chosen as different or not) on overall response strength we calculated the global field power (GFP) which is a reference- and topography-independent measure [12,25]. To determine the time points when activity was significantly different for ‘chosen’ and ‘non-chosen’ epochs we used a permutation test that controls for the family-wise error rate (FWER) associated with the multiple comparisons [26]. Simulated GFP differences were generated over 5000 iterations by randomly assigning epochs to be chosen or non-chosen. The distribution of the minimum and maximum t-statistics associated with each of the 5000 comparisons was used to generate the criterion t-statistic at α = 5%. We also followed the methods described by Murray et al. [25] and used by Bernasconi et al. [12] to assess topographic dissimilarity between chosen and non-chosen epochs, regardless of the overall electric field strength and baseline. Like the GFP, topographic dissmilarity is reference-independent. The measured dissimilarity was compared to the simulated dissimilarity generated over 5000 iterations. We accepted only topographic differences for which p < 0.05 over at least 5 consecutive time-points (10 ms) to be significant. It is not possible to fully control for the FWER using a permutation test because topographic dissimilarity does not use a t-statistic. We followed instead the convention set by Bernasconi and colleagues [12] to be able to compare the results of both studies. Given this caveat the dissimilarity results might be inflated.

## Results

### Analysis of behavioral responses

The use of identical stimuli in a discrimination task raises the possibility that listeners used non-perceptual strategies when performing the task. Two types of bias were analyzed with respect to the dataset: (i) stationary bias, reflecting interval preference; and (ii) non-stationary (dynamic) bias, reflecting serial effects when the choice depends on the previous trial. We further investigated the effect of the random feedback on both of these types of bias.

Although individuals varied considerably, there was an overall bias towards choosing interval 1 (37.65%) more often than interval 2 (33.75%), which was chosen more often than interval 3 (28.60%) (χ^2^ test: p < 0.001 for main effect and all *post-hoc* comparisons). An interval-selection preference was observed in 15 of the 17 listeners. The random feedback provided by the software corresponded with the listeners’ choice on 32.93%, 33.55% and 33.51% of interval 1, 2, and 3 button-presses, respectively. The listeners’ response preference was unrelated to the feedback provided; the distribution of responses was significantly different from the distribution of feedback intervals (χ^2^ test: p << 0.001).

Listeners also had a preference for alternating between different responses to a larger extent than would be predicted by chance, resulting in a significant overall serial effect (χ^2^ test: p < 0.001). This was also significant in 12 of the 17 individual listeners. These results were validated by a simulation showing that a random permutation of responses within subjects makes the serial effects disappear. An over-proportionate alternation between intervals could result from the relatively low rate of “correct” feedback (this would be kept at about 70-80% in most psychophysical studies). However, there was no evidence of an effect of the feedback on the serial effects in the selection of response interval (χ^2^ test: p > 0.54 for all intervals). Both types of bias were thus found to be present in the dataset, but neither was affected by the feedback. According to SDT, response biases affect the placement of the decision criterion. The existence of these non-sensory top-down influences suggests any pre-decision physiological differences we observe may be underestimated because the criterion may have dynamically shifted during the experimental session.

Although the task was not speeded, reaction times (RTs) were calculated for each interval using 5% trimmed means to avoid the influence of extreme outliers. Mean RTs were 942 ms for interval 1 (median: 831 ms; range 553–1518 ms), 978 ms for interval 2 (median: 850 ms; range: 472–1711 ms), and 859 ms for interval 3 (median: 755 ms; range: 360–1587 ms). Mean RTs did not differ significantly between intervals (ANOVA: F(2,48) = 0.53; p = 0.59).

### Event-related potentials (ERPs)

**Figure 2:**
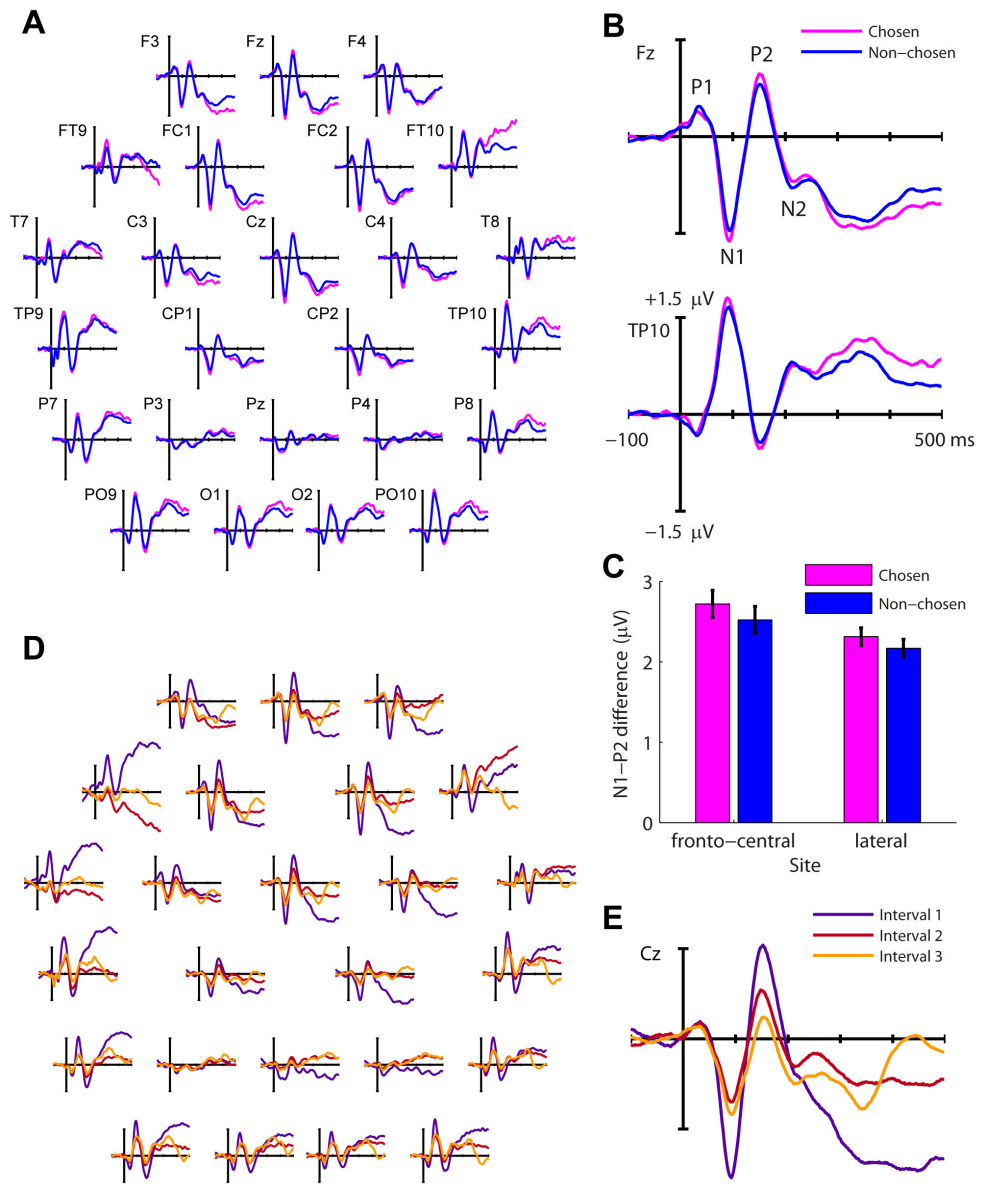
Grand average event-related potentials (ERPs). (A) ERPs associated with chosen (magenta) and non-chosen (blue) stimuli, averaged across intervals and listeners, shown on selected electrodes plotted to approximate topography. (B) Enlarged plots for ERPs to chosen and non-chosen stimuli on a fronto-central electrode (Fz; top) and a lateral electrode (TP10; bottom) show the polarity reversal typically associated with generators in the auditory cortex on the superior temporal plane. (C) Marginal means associated with the absolute N1-P2 mean amplitude differences for chosen (magenta) and non-chosen (blue) stimuli at fronto-central and lateral sites. Error-bars denote the within-subject confidence intervals for the chosen – non-chosen difference after removing inter-subject variability (see 58). (D) ERPs for interval 1 (purple), 2 (red) and 3 (orange), averaged across chosen and non-chosen stimuli and listeners. (E) Enlarged plot for the ERPs in each interval on the midline central electrode (Cz).

Several negative and positive peaks were identified in the ERP waveforms (Figure 2A,B). An early positive deflection peaking at 40 ms over frontal channels was identified as the P1. The N1 peaked over fronto-central channels at ~95 ms, followed by a positive peak identified as P2 which peaked at ~160 ms. The N1 and P2 showed a similar scalp distribution, including a polarity reversal between frontal and lateral sites, confirming sources on the superior temporal plane [27,28]. Following the P2 we observed a large and slow negative wave. It temporally and topographically partially overlapped a negative deflection peaking over left fronto-central channels at ~220 ms, identified as the N2b subcomponent of the N2 wave (see 24), which appeared as a bump on its descending slope.

The lack of a P3 wave, a positivity recorded in the 300-600 ms time-window at fronto-central sites (P3a) during involuntary target (novelty) detection or parietal sites (P3b) during voluntary target detection (reviewed in 29) is surprising. It is quite possible that this component was swamped by the slow negative wave we observed, peaking at 300-400 ms (Figure 2B). A similar slow negative wave in the absence of a P3 was observed for tone discrimination by Ben-David and colleagues [24]. Rohrbaugh et al. [30] also observed a slow negative wave in response to attended, task-relevant auditory stimuli, sometimes in the absence of a P3. However, in Rohrbaugh et al.’s study this wave peaked ~600 ms after stimulus onset and returned to baseline about 2 seconds later, the timescale of our trials (including the response time and inter-trial period) but not of individual interval epochs. The interval-by-interval ERPs in Figure 2D and E shed some light on the time-course of this wave if we consider that each interval was individually baseline-corrected to the 100 ms preceding each stimulus. The onset of the wave immediately followed the P2 in interval 1, but interval 2 and 3 ERPs overlapped with its slowly ascending slope as it returns to baseline. The N2b and possibly a P3 can be seen at the vertex channel (Cz) in both intervals 2 and 3 (Figure 2E). An additional fronto-central negative peak was observed at ~330 ms in interval 3. Since we were more interested in how early activity differences affect the behavioral choice, a full analysis of the adaptation effects is outside the scope of this paper but we include topographies at major points of difference between the three intervals in Figure S2 online.

### Differences in the N1-P2 region

We expected differences in internal noise to be observed at an early stimulus encoding stage. Indeed, the N1 peak amplitude appeared larger for chosen stimuli at both fronto-central and, with reversed polarity, at lateral sites (Figure 2B). We chose to analyse the N1-P2 difference rather than the N1 amplitude because chosen and non-chosen epochs as well as the different intervals may have different baselines. We calculated mean peak amplitudes over 25 and 30 ms time windows for N1 and P2 respectively, and subjected the difference to an electrode × interval × choice ANOVA (with hemisphere added as a factor in the analysis over lateral sites; see Methods section for details). Chosen responses had significantly larger N1-P2 difference over fronto-central electrodes than non-chosen responses (Figure 2C: F(1,16) = 12.5; p = 0.003; η_p_^2^ = 0.44). The chosen response N1-P2 difference was also significantly larger over the lateral sites (F(1,16) = 14.9; p = 0.001; η_p_^2^ = 0.48), with no difference between left and right hemisphere (F(1,16) = 2.32; p = 0.15; η_p_^2^ = 0.13).

The N1-P2 difference was larger in the first interval compared to both second and third intervals (Figure 2D and E; frontal site: F(2,32) = 54.9; p < 0.001; η_p_^2^ = 0.77; lateral site: F(2,32) = 50.2; p < 0.001; η_p_^2^ = 0.76), reflecting short-term stimulus adaptation. Critically, there was no interaction between interval and choice (frontal site: F(2,32) = 1.78; p = 0.19; η_p_^2^ = 0.10; lateral site: F(2,32) = 0.015; p = 0.97; η_p_^2^ = 0.001), suggesting chosen stimuli elicited larger responses in each of the 3 intervals. The presence of the choice effect in each interval also suggests that it does not result from the stationary biases associated with interval selection (see behavioral results above).

The N1-P2 difference magnitude did not vary with channel location, either frontally or laterally (Figure 2A: frontal site: F(3,48) = 0.40; p = 0.60; η_p_^2^ = 0.024; lateral site: F(2,32) = 0.77; p = 0.46; η_p_^2^ = 0.046), and there were no interactions of channel with choice or interval (p > 0.14), suggesting common ERP generators for all stimuli regardless of choice or interval.

### Global field power (GFP) differences between chosen and non-chosen stimuli

**Figure 3:**
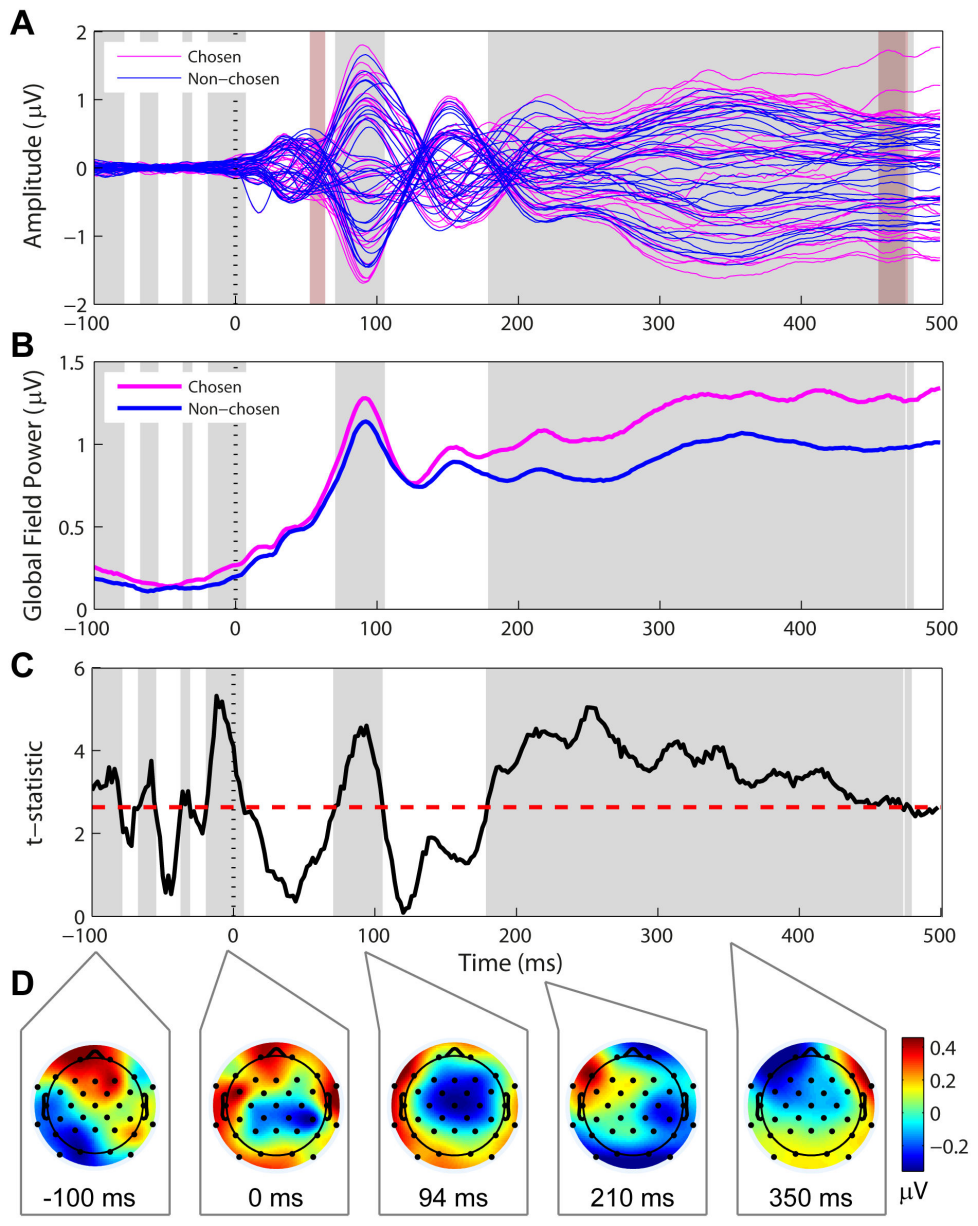
Comparison of ‘chosen’ and ‘non-chosen’ responses: Global field potential (GFP). (A) Single-electrode traces (33 channels) for chosen (magenta) and non-chosen (blue) responses, grand averaged across presentation intervals and subjects. Grey patches demarcate times during which there was a significant difference between GFP for the chosen and non-chosen stimuli (see B and C). The pink patches demarcate areas of significant topographic dissimilarity (see Figure 4). (B) GFP for responses to stimuli ‘chosen’ as different (magenta) and ‘non-chosen’ (blue). These measures are independent of topography differences. Grey patches are as above. (C) Statistical analysis of the GFP: A permutation test was run to control for family-wise error rate (FWER) associated with multiple comparisons across the ERP time-course. The t-statistic associated with the difference between chosen and non-chosen GFP is in black, and criterion t-value controlling for the FWER is marked by the dashed red line. The grey areas demarcate time points at which the t-statistic exceeded the criterion value. (D) Topographic plots showing the difference between chosen and non-chosen activity at various time-points with significant GFP differences. The color bar indicates differences in microvolts.

**Figure 4:**
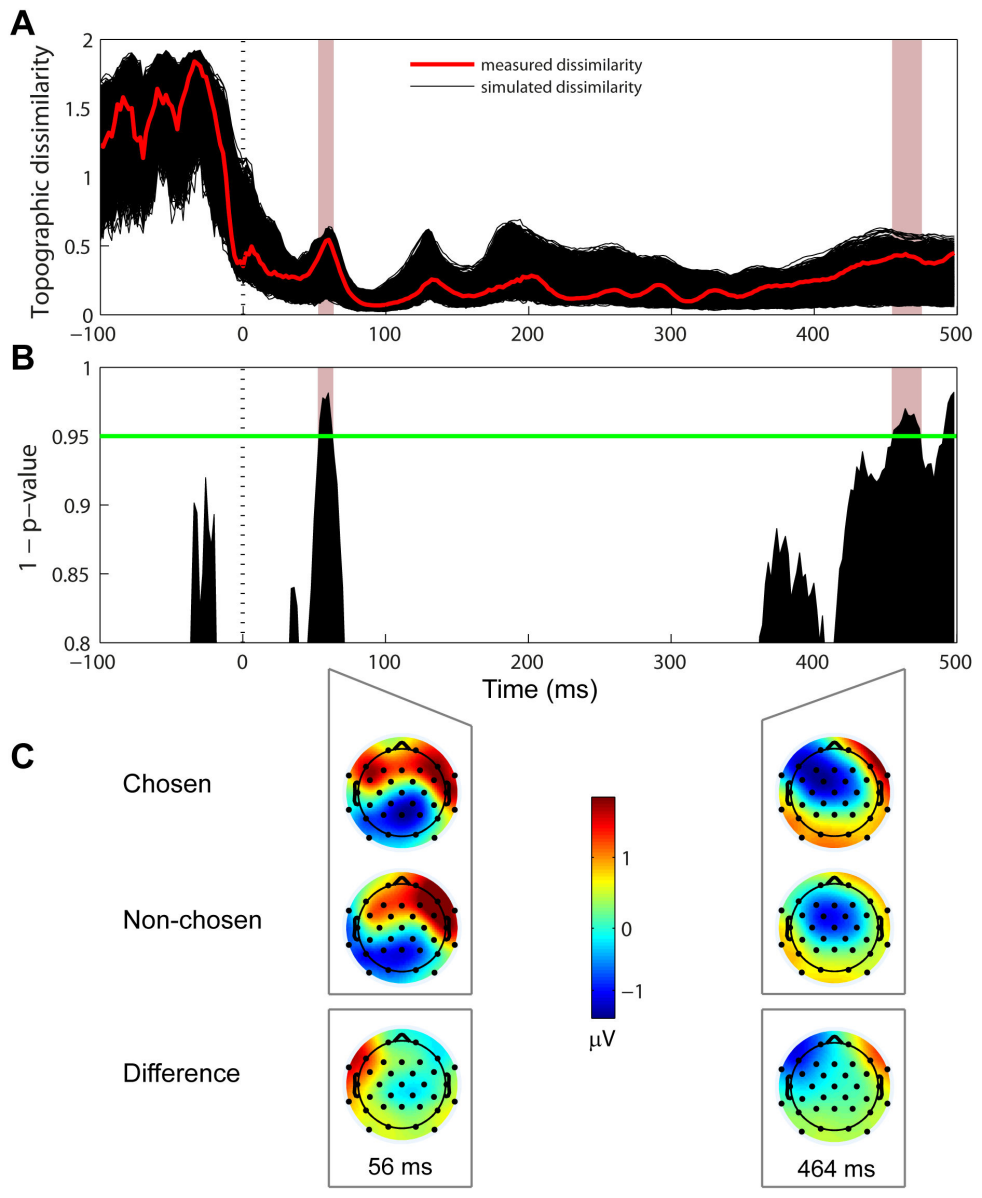
Topographic dissimilarity associated with chosen and non-chosen stimuli. (A) The black traces show the topographic dissimilarity across time with random allocation of chosen and non-chosen epochs (5000 iterations). The measured dissimilarity for our original data set is shown in red. The pink patch demarcates a time region of statistical significance ≥10 ms. (B) Statistical map showing the likelihood of the measured dissimilarity being greater than 95% of the simulated data, as a non-parametric test of significance. The green line marks p = 0.05. (C) Chosen (top), non-chosen (middle) and difference (bottom) topographies associated with the time regions showing a statistically-significant contrast.

Internal noise could have affected processing even earlier than the N1 time window. To check for this possibility, we compared the GFP for chosen and non-chosen stimuli. The GFP measures overall activity level independent of location, so higher overall activity could be indicative of higher internal noise levels. Significantly higher-amplitude GFPs were associated with chosen stimuli in three main time intervals (Figure 3A,B,C) even after controlling for multiple comparisons: the pre-stimulus interval (intermittently from -100 ms to +6 ms), during the N1 ERP from its onset till past its peak amplitude (70-104 ms), and then continuously following the P2 peak till almost the end of the epoch (178-474 ms). Neither the P1 nor the P2 time regions showed significant differences in GFP magnitude (Figure 3B, with statistical analysis in Figure 3C). The topography of the difference between chosen and non-chosen stimuli at various time points of significant GFP difference is shown in Figure 3D. The chosen and non-chosen stimuli have the same topography at these points (The topographic dissimilarity is not significant; Figure 4). Notably, the chosen – non-chosen difference has the same topography as the N1 auditory ERP (see 94 ms time window in Figure 3D), including the polarity reversal between frontal and lateral electrodes indicative of generators in the superior temporal plane [27,28]. The late and prolonged GFP difference may indicate enhanced higher-level processing past the point at which the stimulus was recognized as the ‘target’ rather than internal noise leading up to that point.

### Differences in chosen vs. non-chosen topography

Significant topographic dissimilarity between the two types of stimuli is indicative of different neural generators of activity independently of its overall strength. The topographic dissimilarity between chosen and non-chosen stimuli was significant over a consecutive period >10 ms only at 52-62 ms and 454-474 ms post stimulus onset (Figure 4A,B). The early dissimilarity followed the P1 peak and showed more bilateral activity for chosen epochs compared to right-lateralized activity for non-chosen epochs (Figure 4C, left). The late dissimilarity showed more left-lateralized activity for chosen compared to non-chosen stimuli (Figure 4C, right). The late difference topography (Figure 4C, bottom right) is typical of lateral eye movement. Both of these differences reflected greater activity recorded at left frontal and fronto-temporal electrodes but with reversed polarity, positive for the early and negative for the late time regions (Figure 4C, bottom). Taken together with the GFP results, the pattern of topographic dissimilarity observed here suggests the early increased activity in the pre-stimulus interval as well as during the N1 time window resulted from chosen and non-chosen stimuli invoking the same neural generator(s) but to a different degree. This could be interpreted as resulting from fluctuations due to internal noise resulting in differential encoding of the stimuli.

### End-of-trial processes

**Figure 5:**
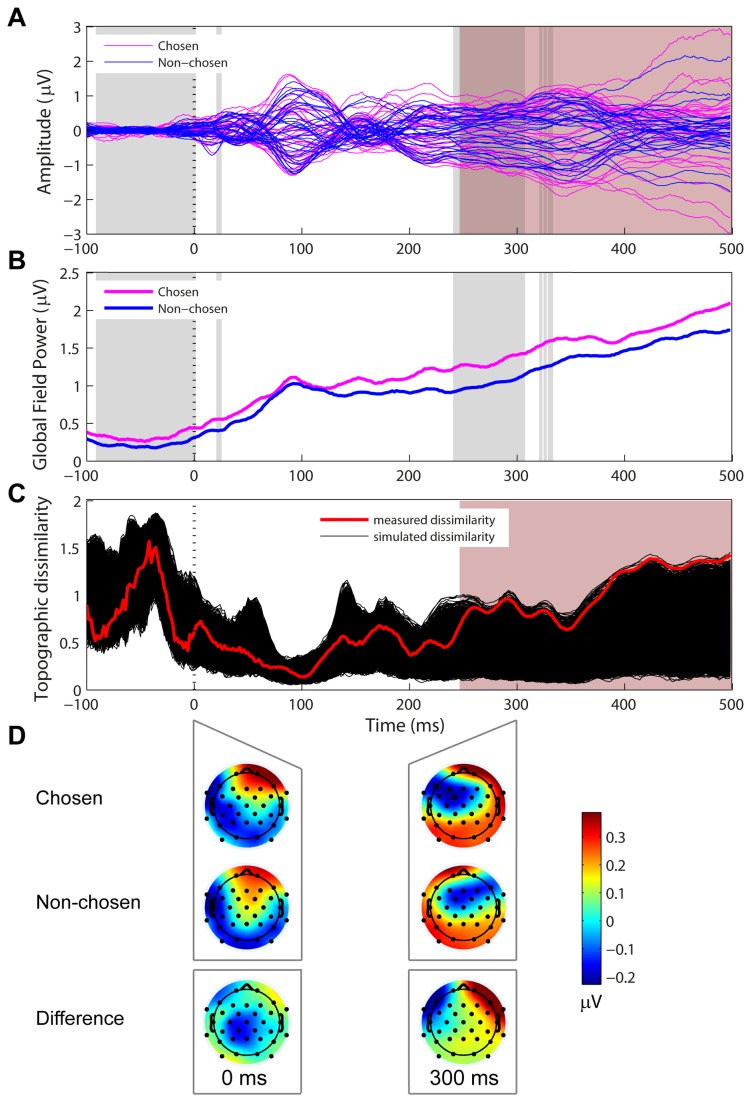
Differences between chosen and non-chosen responses in the final stimulus interval. (A) ERP waveforms for chosen (magenta) and non-chosen (blue) stimuli in interval 3. Each trace represents an electrode channel. The colored underlays match the statistical maps in B and C. (B) Global field potential (GFP) for chosen and non-chosen stimuli in interval 3. The grey patches demarcate regions where the contrast exceeds the criterion t-value calculated using the permutation test to control for multiple comparisons. (C) Topographic dissimilarity for the chosen vs. non-chosen contrast in interval 3. The pink patch demarcates consecutive significance over ≥10 ms. (D) Topographic plots of chosen (top) and non-chosen (bottom) average activity at selected time-points. The color bar shows potential in microvolts.

Although we were primarily interested in early processes leading to the decision, we also examined the time following the presentation of the third stimulus and preceding the response stage of the trial. Figure 5 shows the ERPs associated with interval 3 (Figure 5A), and the GFP (Figure 5B) and the topographic dissimilarity (Figure 5C) associated with the contrast between chosen and non-chosen epochs in this interval. Note that statistical power was reduced here because only one third of the data were used in this analysis – perhaps the reason the N1 difference in GFP was not significant. However, several significant differences were still observed. Firstly, GFP in the period preceding stimulus onset was significantly larger for chosen epochs. Note that non-chosen stimuli here mean that one of the previously presented sounds was ‘chosen’ on those trials. We have no way of determining whether that decision has already been made at that point, but it is clear that choosing the third interval followed increased GFP prior to the third stimulus onset, as was observed in the analysis of all intervals together. Most notable, however, is the topographic dissimilarity between chosen and non-chosen stimuli in this interval (Figure 5C). Responses to chosen stimuli showed more left-lateralized frontal negativity, while non-chosen stimuli showed more right-lateralized frontal negativity (Figure 5D, right panel). The difference topography is typical of lateral eye movements. Although lateral eye movement artefacts were removed as much as possible during pre-processing using ICA, components that contained even residual ERP activity were retained. It is possible that having heard the third tone in the sequence, listeners moved their fixation to the animated character representing the chosen interval prior to making their response. The late topographic dissimilarity in Figure 4C is probably driven by these differences in interval 3. This global dissimilarity overlapped (246-306 ms) with significantly increased GFP for chosen stimuli at a time just prior to the late negative peak (Figure 2E). This conjunction was not present in the data averaged across all three intervals (Figure 2), and we therefore suggest that it is related to end-of-trial processes, possibly reflecting decision-making prior to response.

## Discussion

We found differences in the pre-stimulus, mid- and long-latency responses, as well as at the end of the trial, in the activity produced by stimuli judged to be “different” compared to those judged to be the “same”. The early activity differences (especially as measured by the GFP) are consistent with the signal detection theory (SDT) prediction of internal noise affecting stimulus encoding and representation. These representational differences drive later decision-related processes, resulting in the late differences between chosen and non-chosen stimuli. In the following sections we suggest possible origins of the noise associated with the differences found, and discuss several methodological issues that constrain our interpretation of the results.

### Physical stimuli, internal noise and percepts

The ability to discriminate perceptually between identical stimuli has been linked to the effect of variations in internal noise present in the system during task performance on the internal representations, or percepts, of the stimuli (e.g. [13]). Signal detection theory makes no distinction between low- (e.g. sensory) or high-level (cognitive) sources of internal noise, and both types have been previously reported (e.g. [2,3]). Noise-induced differences in internal representation of physically-identical stimuli are treated by the brain in the same way as differences in physical stimuli. They feed into the decision process, which is ‘unaware’ whether the source of the difference is external or internal. A range of evidence from single-neuron physiology in animals (e.g. [6]) to human neuroimaging (e.g. [9,31]) suggests that perceptual decisions can be driven by an enhanced physiological response to a stimulus compared to the response to physically identical stimuli. The early response variations observed in animals (~50 ms [6]) and humans (~100 ms [12]) are unlikely to be caused by efferent activity from higher-level areas, which is thought to influence the EEG signal only at latencies >200 ms after stimulus onset ( [32], and see a similar argument in [6] about single-neuron responses). Taken together, previous findings suggest that decision-related activity even in the absence of an external stimulus can be traced back to neural activity in low-level areas that represent the stimulus features relevant for the decision. The data reported here partially supports these conclusions, but in the following sections we show that not all the observed variation associated with the decision can be attributed to sensory or low-level origins.

### Pre-stimulus activity

Activity in the 100 ms preceding stimulus onset showed greater GFP preceding stimuli which would be chosen compared to those that would not be chosen. These differences could result from top-down modulation which have been variously referred to as variations in brain state [33] or baseline activation [18,34,35,36]. For example, Wyart and Tallon-Baudry [36] showed that pre-stimulus fluctuations in gamma-band activity correlated with the visual detection of near-threshold gratings. Hesselmann and colleagues [37] showed that fluctuations in pre-stimulus fMRI activation can predict perceptual choices for ambiguous figures (where there is no ‘correct’ response), and Bode et al. [38] likewise showed that pre-stimulus fluctuations in the ERP can predict perceptual decisions on a trial-by-trial basis when the stimuli themselves contained no discriminative information. Although the fluctuations in human studies were observed over a much longer time-scale (several seconds) compared to the time-scale in our experiment (~600 ms), animal studies have shown faster fluctuations in baseline firing rates that were correlated with the stimulus-evoked activity [39,40]. Such fluctuations in ongoing non-sensory activity could nevertheless contribute to the accumulating evidence of the perceived distinction between stimuli leading to a decision [41].

The strength of pre-stimulus activity could also be indicative of a ‘decision bias’ – a predisposition to choose a particular response prior to stimulus onset [38,42]. Despite using very different methodologies, both Shadlen and Newsome [42] and Bode et al. [38] observed a bias towards repeating a previously rewarded response which effectively shifted the decision criterion closer to that choice. While we observed an above-chance tendency to alternate behavioral responses independent of reward feedback, we cannot rule out the possibility that bias contributed to the pre-trial activity variation (prior to interval 1). How bias might affect activity prior to interval 2 or 3 is further complicated by the activity history of preceding interval(s). We think it unlikely that listeners could maintain a pre-trial selection strategy (e.g. “I chose interval 1 in the last trial, so I should choose interval 2 or 3 in this one”) for the length of the experimental session (800 trials) and during the relatively short (~700 ms) response-to-next-trial-onset time. No listeners have reported using a non-perceptual strategy during debriefing (in fact, they mostly claimed the task was “extremely difficult”), but that in itself does not preclude the existence of a pre-stimulus decision bias. Since choice or interval selection bias (both stationary and dynamic) is present in some perceptual tasks near threshold even when the stimuli are physically different (unpublished data from our lab), such bias can be considered a source of “internal” noise, albeit of a cognitive origin.

### Early topographic modulation

An early (~50 ms after stimulus onset) and brief (10 ms) period of topographic dissimilarity between chosen and non-chosen stimuli showed more bilateral frontal positivity in the former that did not coincide with the P1 peak (Figure 4C). The timescale agrees with Britten et al.’s [6] finding of motion direction-dependent activity that matched monkeys’ behavioral decision even in the absence of an actual motion signal. However, it is unclear whether and how a topographic modulation on the gross spatial scale measurable by EEG can be related to the variation in trial-by-trial activity of single neurons in local networks.

Perhaps more relevant are the findings of Bernasconi et al. [12] of topographic differences between the stimuli chosen as “targets” and the non-chosen stimuli in two auditory discrimination tasks (pitch and duration discrimination) using identical sounds. These differences appeared later than ours, at ~100 ms and past the N1 peak, and, at least for the duration discrimination task, appear to have a similar topography. Although Bernasconi and colleagues linked these topographic differences to random fluctuations of activity at specific time points, it is unclear how these relate to different perceptual decisions regarding the scaled parameters of pitch or duration. Although topographic modulation was previously observed in low-level auditory cortical regions in a human fMRI experiment using pitch [43], it depended on listeners being asked to attend to different dimensions of the same stimulus, not different values along the same dimension as in Bernasconi et al. [12] and the present study.

The early topographic modulations are also difficult to reconcile with the SDT understanding of the role of internal noise in the discrimination of identical stimuli [13]. SDT regards internal noise as fluctuations in the activity in the discriminated variable dimension or feature (e.g. pitch), which, given the scale of the tonotopic mapping in early sensory areas, should not be associated with different topographies on the spatial scale that could be measured by EEG or even fMRI. How these topographic modulations are related to internal noise sources that drive the perceptual decision is therefore unclear.

### N1 – P2

Based on the SDT concept of internal noise we predicted that fluctuations in noise level would affect early stimulus encoding, distinguishing between the identical stimuli and hence driving perceptual decisions. In line with this prediction, our results show both greater GFP for chosen than non-chosen stimuli in the N1 time window and greater N1-P2 differences for chosen stimuli, driven by the larger N1 mean amplitude to the sound perceived as different. The N1 wave is generated in the superior temporal gyrus (auditory cortex) in response to sound [28] and has been suggested to reflect stimulus encoding [44]. It is subject to adaptation, reducing in amplitude with stimulus repetition [45,46], as we have also observed. N1 amplitude has previously been found to depend on the frequency or intensity disparity (increment or decrement) between two sounds [47]. The enhancement we observed in the N1-P2 difference mimics this characteristic. The topography of the chosen – non-chosen difference in GFP is indicative of internal noise originating in sensory auditory cortex.

The N1 is sensitive to attentional modulation, showing an enhancement to attended compared to ignored stimuli (e.g. [48]). We therefore considered the possibility that the N1 enhancement we observed resulted from one interval being selectively attended while others were ignored. However, previous findings showed that whereas the N1 amplitude was enhanced, the P2 amplitude was suppressed to attended compared to unattended stimuli ( [27,49,50]; see review in [51]). We might therefore have expected the N1-P2 difference not to be modulated by attention, as these two effects could cancel one another or at least diminish the difference. The GFP was still larger for chosen than non-chosen stimuli in the P2 time window, albeit not significantly so, contrary to what would be expected from selective attention.

Animal studies that looked at attentional modulation of perceptual choice signals in single neurons have all used paradigms where attention was directed towards a specific spatial location, stimulus or stimulus feature (see review in [52]). This was not the case in the present study; to perform the task listeners needed to attend to all stimulus intervals and make a comparison. While we certainly do not discount the possible effects of attention as a source of internal noise, we suggest that it was either exogenous attention, captured by the perceived oddball stimulus, or fluctuations in attention that were unrelated to the timing of the task, but that may have affected baseline activity (see 53).

### Late and end-of-trial differences

The GFP showed later sustained differences between chosen and non-chosen stimuli following the P2 peak (from about 175 ms post stimulus onset) almost to the end of the epoch. These differences included the N2 peak time window, which has been implicated in decision-making processes of stimulus classification or target selection for response [54,55]. We recorded a small N2b wave, thought to be elicited by a template mismatch, or a deviation from a stored representation of the stimulus [56]. The N2b reflects ‘voluntary’ processing [56] rather than exogenous attentional orienting to an oddball stimulus. It is possible that internal noise may cause perturbations (reflected in earlier, exogenous potentials such as the N1) that, when large enough, result in a deviation from a stored representation, or perceptual anchor [57], leading to an enhanced N2b. This component could therefore reflect the classification of the chosen stimulus as “different” (i.e. its identification as the “target”), leading to its selection for response (see 24). However, this possibility requires experimental examination.

The differences (both in GFP and topography) observed at the end of the trial (following the third stimulus) may reflect the actual perceptual decision or response choice, though we do not rule out that choice related activity was already present following the actual ‘chosen’ stimulus and not only delayed until the end of the trial. In the third interval the increased GFP coincided with topographic differences that are typical of lateral eye movements. These may indicate that a choice has been made and possibly reflect choice-related lateral eye movement to fixate the character representing the chosen interval in anticipation of the feedback reward (an animation of that character).

### Methodological considerations

We interpreted our findings of choice-related variation in early and late activity as reflecting internal noise that drives the perceptual decision, respectively. Here we consider two methodological issues that could potentially confound this conclusion. We first considered the possibility that the perceptual decision in each trial was random, or unrelated to the neural activity differences. Although we are unable to demonstrate the relationship between activity and decision on a trial-by-trial basis because the unspeeded, 3-interval design did not allow us to determine the point at which the perceptual decision was made, the across-trial averages indicate the chosen intervals tended to have greater amplitudes. Given the power associated with our non-parametric statistical analyses, it is highly unlikely that such significant and consistent differences between two portions of the same data set were unrelated to the behavioral decision.

Secondly, the adaptation effect we observed across trial intervals means the neural response to the first interval was always the largest, and this difference was much larger than the chosen – non-chosen effect. Indeed, this could have contributed to the bias we observed in choosing interval 1 more often than either other interval. The fact that it wasn’t always perceived as different suggests that listeners do not merely rely on stronger activity within a trial, but instead internally calibrate or normalize the first interval activity by comparison with the other two, or develop interval-specific perceptual anchors that allows them to compare each interval activity to the same interval in other trials.

## Conclusions

We interpret the pre-stimulus and early activation differences between chosen and non-chosen tones to reflect internal noise, whether of top-down (fluctuations in attention, variations in brain state) or bottom-up (stochastic firing in sensory neurons) origin. These variations contribute to the formation of a decision variable and are reflected in both later activity differences and the behavioral choice.

## Supporting Information

Figure S1 Screenshot of a typical monitor display used in presenting the behavioral task. Three identical characters were presented, equally spaced horizontally across a 17″ computer screen. The characters were sequentially animated from left to right to briefly open their mouths to coincide with tone presentation (see example of middle character above). Following a “correct” response, positive feedback took the form of the chosen character waving its arms up and down briefly. The characters and background scenery changed after every 100-trial block. Participants were seated approximately 1.1-1.2 m from the screen. There was no fixation point and eye movements were not controlled for.
(TIF)

Figure S2 Topographies for time points (rows) at which the activity in the 3 intervals (columns) differed. (TIF)
