# Supplementary figures and images for: Human Decision Making Based on Variations in Internal Noise: An EEG Study
Source: PLoS One. 2013 Jul 1;8(7):e68928. doi: 10.1371/journal.pone.0068928 (PMC3698081; doi:10.1371/journal.pone.0068928)

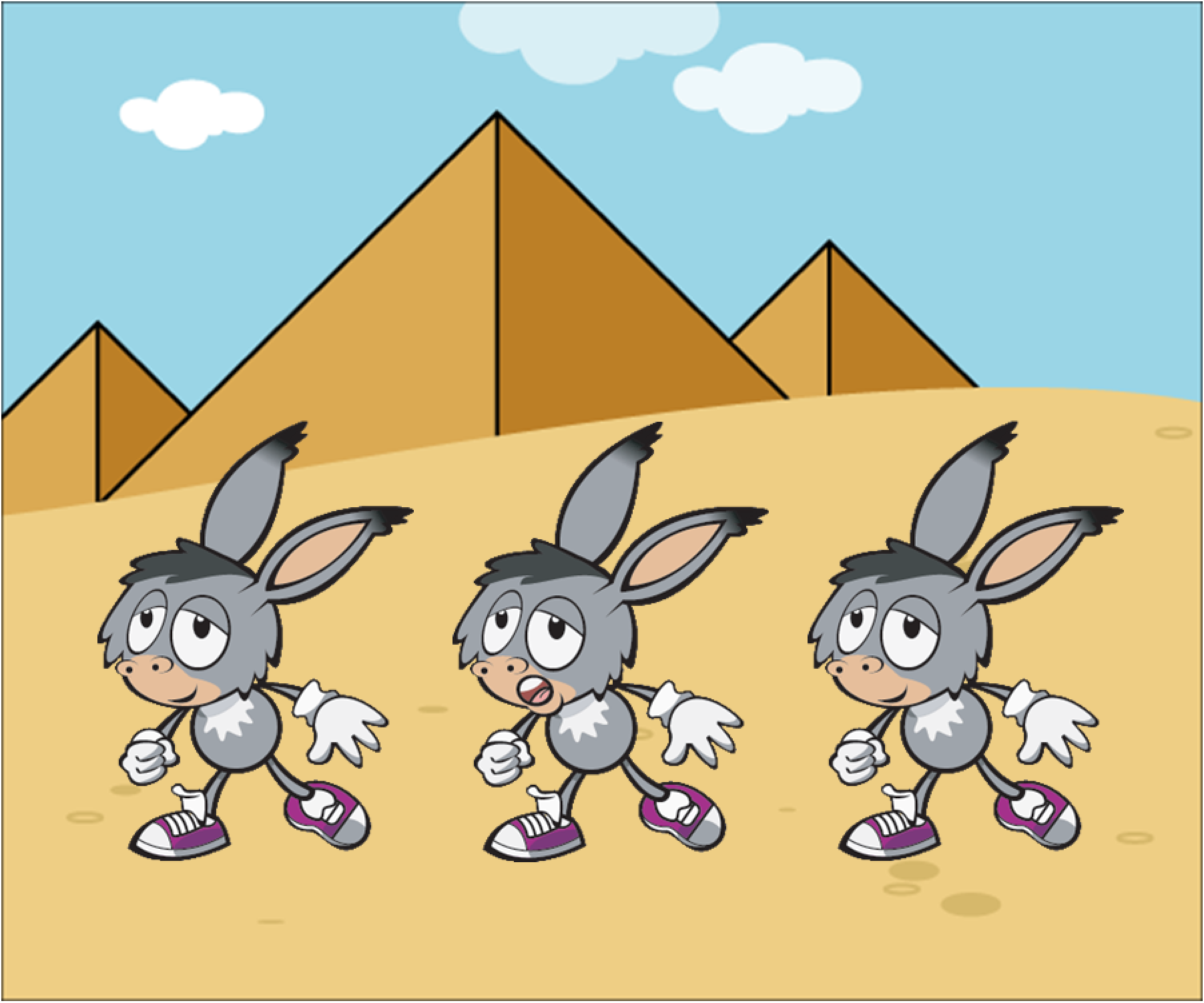

Supplement: Figure S1 — Three identical characters were presented, equally spaced horizontally across a 17″ computer screen. The characters were sequentially animated from left to right to briefly open their mouths to coincide with tone presentation (see example of middle character above). Following a “correct” response, positive feedback took the form of the chosen character waving its arms up and down briefly. The characters and background scenery changed after every 100-trial block. Participants were seated approximately 1.1-1.2 m from the screen. There was no fixation point and eye movements were not controlled for. (TIF) [file pone.0068928.s001.tif]

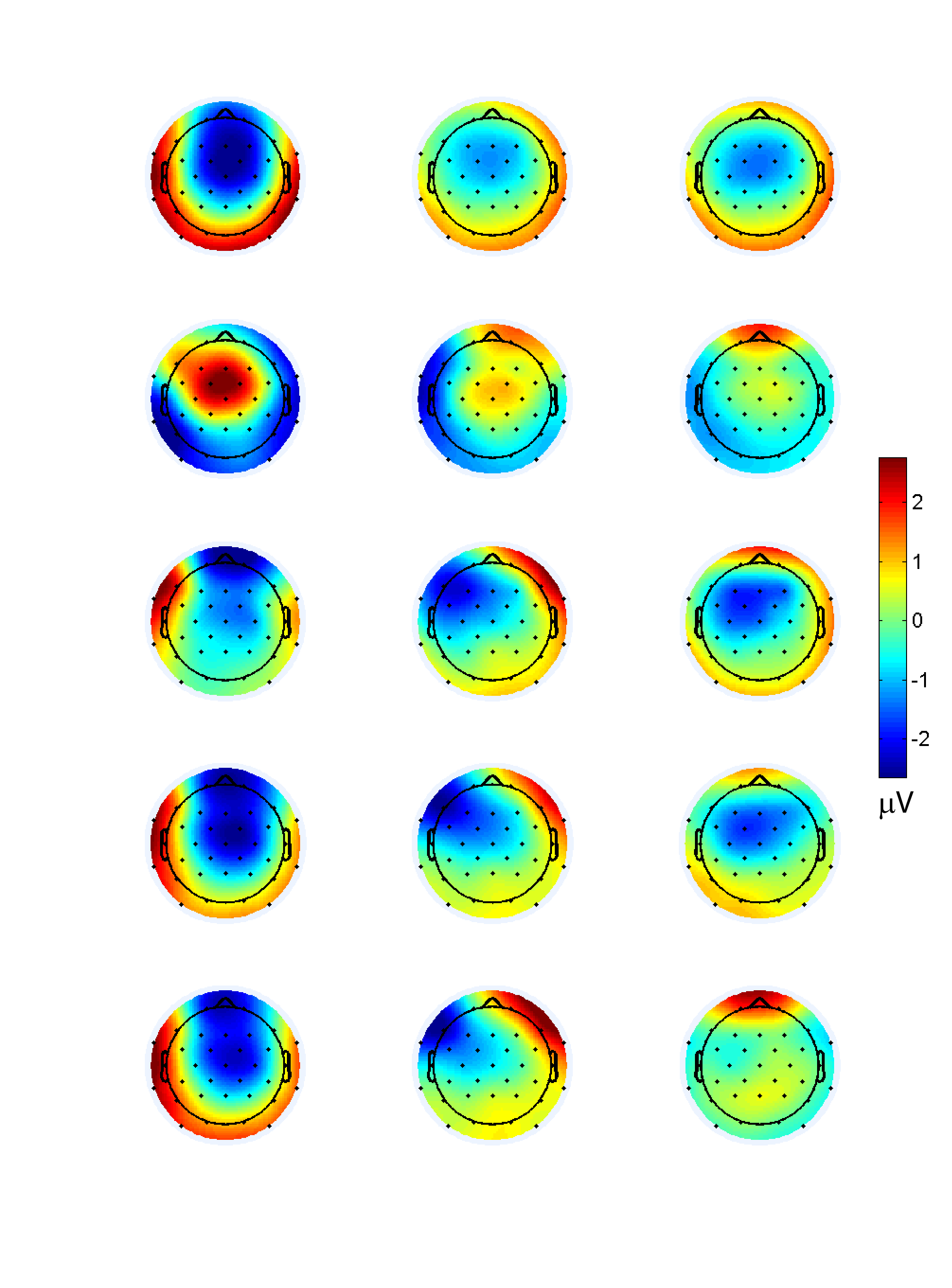

Supplement: Figure S2 — (TIF) [file pone.0068928.s002.tif]
